# Downregulation of proinflammatory cytokines in HTLV-1-infected T cells by Resveratrol

**DOI:** 10.1186/s13046-016-0398-8

**Published:** 2016-07-22

**Authors:** Maria Pia Fuggetta, Valentina Bordignon, Andrea Cottarelli, Beatrice Macchi, Caterina Frezza, Paola Cordiali-Fei, Fabrizio Ensoli, Stefania Ciafrè, Francesca Marino-Merlo, Antonio Mastino, Giampietro Ravagnan

**Affiliations:** Institute of Translational Pharmacology (IFT), National Research Council (CNR), Via Fosso del Cavaliere 100, 00133 Rome, Italy; Laboratory of Clinical Pathology and Microbiology, San Gallicano Dermatologic Institute, Via Elio Chianesi, 53, 00144 Rome, Italy; Department of System Medicine, University of Rome “Tor Vergata”, Via Montpellier 1, 00133 Rome, Italy; Department of Biochemical Science and Surgery, University of Rome “Tor Vergata”, Via Montpellier 1, 00133 Rome, Italy; Department of Chemical, Biological, Pharmaceutical, and Environmental Sciences, University of Messina, 98166 Messina, Italy

**Keywords:** Resveratrol, HTLV-1, Inflammatory cytokines

## Abstract

**Background:**

Human T-cell leukemia virus (HTLV-1) is a lymphotropic retrovirus associated to adult T cell leukemia (ATL) and to non-neoplastic inflammatory conditions affecting the central nervous system, lung or skin. The inflammatory disorders associated to HTLV-1 are mediated by different proinflammatory cytokines as IL-1α, IL-6, TNF-α. The release and the role of IL-17 is still debated. Aims of this study were to analyze IL-17 induction by HTLV-1 infection and to determine whether resveratrol (RES) is able to down regulate the pathway of cytokines production either in HTLV-1 chronically infected MT-2 cell line or in human CD4+ cells infected in vitro with HTLV-1.

**Methods:**

MT-2 and HTLV-1 infected CD4+ cells were analyzed for proinflammatory cytokine production before or after RES treatment. The concentrations of IL-17, IL-1α, IL-6, and TNF-α were measured in cell culture supernatants by ELISA and SearchLight™ technology. The IL-17 mRNA expression was evaluated by RT-PCR. NF-kB activation was detected by non-radioactive, Electro Mobility Shift Assay (EMSA). HTLV-1 RNA expression was detected by Real-time-PCR (RQ-PCR).

**Results:**

We found that RES is capable of inducing a dose-dependent inhibition of IL-1α, IL-6 and TNF-α production in vitro and can down regulate the expression of IL-17 at both mRNA and protein levels in HTLV-1 infected cells. This effect was associated with a dose-dependent inhibition of the of the nuclear factor kappa-B (NF-kB) activity. Conversely, RES did not apparently affect HTLV-1 proliferation.

**Conclusions:**

These results support the anti-inflammatory properties of RES, suggesting that it might be a useful therapeutic agent for the treatment of HTLV-1 related inflammatory diseases.

## Background

The HTLV-1 virus infects approximately 10–20 million people worldwide [1]. Firstly identified as the etiological agent of ATL it was successively associated with progressive neurological disorders such as the HTLV-1-associated myelopathy/tropical spastic paraparesis (HAM/TSP), a chronic inflammatory disease of the central nervous system [2]. Other non-neoplastic inflammatory diseases such as uveitis, Sjögren syndrome,arthritis polymyositis and periodontitis, have been also recently associated to HTLV-1 infection [3].

The HTLV-1 associated disorders HAM/TSP is characterized by spontaneous proliferation of CD4+ lymphocytes that harbor a provirus, which appear capable of promoting an active expansion of infected T cells. Inflammatory cytokines have been involved in the pathogenesis of HAM/TSP and high levels of IL-2, IFN-γ, and TNF-α appear to contribute to neurological damage in HAM/TSP patients [4]. HTLV-1 is capable to infect CD4+ T and CD8+ T cells in vitro [5] and to immortalize healthy CD4+ T cells that undergo to an active expansion and proliferation [6]. Although HTLV-1-associated disorders have been extensively studied, the mechanism by which HTLV-1 induces inflammatory conditions is still unclear. Moreover, the exact cellular and molecular events underlying the induction of chronic inflammation during infection is not completely understood.

The HTLV-1 provirus encodes a trans-acting factor (Tax) that operates in association with the activating transcription factor/CRE binding protein (ATF/CREB) to enhance virus transcription. Tax induces the expression of both viral and several cellular genes involved in different pathways [7]. Interestingly, HTLV-1 Tax has potent effects on infected T cells, including activation of nuclear factor NF-kB with a subsequent enhancement of cell proliferation and expression of different genes [8]. Peripheral blood mononuclear cells (PBMC) naturally infected by HTLV-1, such as in HAM/TSP disease, precociously express intracellular Tax protein and interferon-γ (IFN-γ) in mitogen- stimulated short term culture, while the expression of IL-2 and TNF-α requires longer culture time [9, 10]. In agreement with these experimental data, a recent study showed that an increase of IFN-γ and TNF-α is involved in the tissue damage observed in clinical complications of HTLV-1 infection [11]. In HTLV-1-infected T cells, Tax up-regulates the expression of IL-17 and of its mRNA [12] and lymphocytes from a HAM/TSP patient have been shown to express in vitro detectable levels of Tax and IL-17 mRNA [13]. Thus, clinical and experimental data suggest that HTLV-I infected cells are prone to produce inflammatory cytokines which might in turn activate resident immune cells and amplify production of pro-inflammatory mediators within tissues in HTLV-1 infected patients. In light of this, individuals considered at risk of HTLV-1 related chronic inflammation could be optimal candidates to be treated with immunomodulatory agents as an adjuvant therapy.

Resveratrol (RES), or trans-3,5,4’-trihydroxystibene, is a polyphenolic, antifungal natural phytoalexin found in grapevines (Vitis vinifera) and a variety of other plants. RES has been shown to possess potent antioxidant and anticancer properties and has been investigated for its immunomodulating and anti-inflammatory capabilities [14–16]. In our previous studies [17] we have demonstrated that RES is able to modulate the expression of cytokines in CD4+ and CD8+ T cells stimulated with anti-CD3/anti-CD28, and could produce an inhibitory effect on IFN − γ, IL-2 and IL-4 production when used at high concentration. We have previously described [18] a significant inhibitory effects of RES, as well as of its derivative polydatin product, on the production of IL-17 in a model of cell inflammation in vitro. In addition, recent studies have reported that RES is active in a wide variety of virus infection [19, 20] revealing strong antiviral activity and an inhibitory effect on proinflammatory cytokine secretion in enterovirus 71 (EV71) infected cells [21].

The capability of RES in blocking nuclear factor kappa-B (NF-kB) mediated catabolic activity [14] makes him a promising therapeutic agent for the treatment of inflammatory conditions suggesting its potential clinical relevance in the therapy of inflammatory diseases.

This study was aimed at evaluating the in vitro effect of RES on the production of proinflammatory cytokines, including IL-17, in a HTLV-1 chronically infected cell line (MT-2), and in HTLV-1 infected immortalized human CD4+ T lymphocytes.

## Methods

### Reagents

Natural RES was extracted and kindly supplied by Dr. Fulvio Mattivi, (Fondazione Edmund Mach, IASMA) Italy. The purity of the compound tested by HPLC-MS, UV, and NMR was higher than 99 % (method patented, Ravagnan et al. 2001, PCT/IB01/00983) [22]. RES was dissolved in dimethyl sulfoxide (DMSO, Sigma Chemical Co., St. Louis, USA) at 100 mM stock solution. The stock solutions were stored at −80 °C and diluted in culture medium just prior to use.

### Cells

MT-2 cells, a HTLV-1 producing cell line derived from cord blood lymphocytes infected in vitro with HTLV-1 from infected patients [23], were grown in RPMI-1640 with 20 % heat-inactivated fetal calf serum (FCS- Hyclone Laboratories, Logan, UK), (Hyclone Europe, Cramlington, UK) 2 mM L-glutamine and penicillin-streptomycin (Life Technologies Ltd., Paisley, Scotland), hereafter referred to as complete medium (CM) and split weekly.

To assess HTLV-1 infection in vitro, lymphocytes were isolated from peripheral blood mononuclear cells (PBMC), obtained from healthy donors. Buffy coats were purified by Ficoll-hypaque gradient centrifugation (Sigma-Aldrich Corp) and CD4+/CD3+ cells were isolated by separation through immunomagnetic beads [6]. HTLV-1 infection was obtained by co-cultivating CD4+ lymphocytes with lethally irradiated (120 Gy, from a cesium Gamma cell 1000, Atomic Energy Canada) HTLV-I producing MT-2 cells at an acceptor: donor ratio of 3:1. Co-cultures were maintained in the presence of recombinant IL-2 (rIL-2, Hoffman-la Roche, Basel Switzerland) and split weekly The presence of HTLV-1 proviral DNA and of Tax/Rex mRNA in infected cells was determined at several time during the culture.

Cell growth and total number of viable cells were determined by the MTT assay and by the Trypan blue dye exclusion test, respectively, according to standard procedures.

### Cytokine production and analysis

MT-2 and HTLV-1 infected CD4+ cells were harvested, counted, suspended in CM and seeded into 24-well tissue culture plates (Falcon) in 1 ml/well at a concentration of 1x10^5^ cell/ ml. The plates were incubated at 37 °C in a 5 % CO2 humidified atmosphere for 30 min, 1, 3, 6, 12, 24, 48 or 72 h. Each culture was set in triplicate. At each time, cells were analyzed for proinflammatory cytokine production. Culture supernatants of MT-2 and HTLV-1 infected CD4 + cells were collected as control for cytokine detection.

The concentrations of IL-1α, IL-6, and TNF-α were measured in undiluted cell culture supernatants in triplicate by multiplex enzyme-linked immunosorbent assay based on SearchLight™ technology (Thermo Fisher Scientific Pierce Searchlight Products, Woburn, MA). The amount of signal produced is proportional to the amount of each protein in the sample detected with a cooled CCD camera. The assay [24] is capable of detecting target proteins at pg/ml concentrations.

To detect IL-17 production, a quantitative sandwich enzyme immunoassay (R&D Systems). technique specific for natural and recombinant human protein was used. According to the manufacturer, the detection limit of the assays was approximately 15.0 pg/ml.

### RES treatment

MT-2 and HTLV-1 infected CD4+ cells were harvested, counted, suspended in CM and seeded into 24-well tissue culture plates (Falcon) in 1 ml/well volume at a concentration of 1x10^5^ cell/ml. Cells were incubated in the presence or in the absence of RES at final concentrations of 0.625, 1.2, 2.5, 5, 10, 20, 40 μg/ml, or with DMSO alone as control. The plates were incubated for 0.5-48 h at 37 °C in a 5 % CO2. After each treatment, cell growth and viable cells were determined by Trypan blue exclusion test(data not shown). To evaluate the duration of the inhibitory effect of resveratrol on IL-17 production, MT-2 cells (2.5 10^4^ cell/ml) were treated with 20–40 μg/ml RES for 48 h and then washed and re-seeded into 24-well tissue culture plates at a concentration of 1x10^5^ cell/ ml. Culture supernatants were collected for IL-17 detection (ELISA kit Quantikine, h-IL-17 immunoassay, R&D Systems, Minneapolis, USA).

### IL-17 mRNA expression through RT-PCR

Total RNA was extracted from MT-2 and HTLV-1 infected cells using the RNeasy mini kit (Qiagen), according to manufacturer’s instructions. RNA was extracted from either non-treated cells or from cells treated with 20 μg/ml of RES. Reverse transcription-PCR was carried out with the PCR CORE kit (Applied Biosystem, Roche) using random hexamers and 1 μg total RNA for first-strand synthesis. The cDNA encoding IL-17 was amplified using the oligonucleotides IL-17 forward (5’-ATGACTCCTGGGAAGACCTCATTG-3’) and IL-17 reverse (5’- CTTAGGCCACATGGTGGACAATCG −3’) by 40 PCR cycles of 95° for 30 s, 70° for 30 s, and 72° for 30 s. As control, the constitutive human GAPDH cDNA was PCR amplified using the oligonucleotides GAPDH forward (5’-ATGGTTTACATGTTCCAATATGATTCC-3’) and GAPDH reverse (5’-CTTACTCCTTGGAGGCCATGTGGG-3’). The amplified products were electrophoresed on 2 % agarose gel in 1× TAE (Tris acetate EDTA).

### Detection of HTLV-1 RNA expression by Real-time-PCR (RQ-PCR)

RNA isolation was performed using EuroGOLD total RNA kit (Euroclone), according to the manufacturer’s instructions. For RQ-PCR, 250 ng of total RNA from each sample was reverse transcribed in a total volume of 20 μl using the high-capacity cDNA reverse transcription kit (Applied Biosystems, CA, USA), according to the manufacturer’s instructions. Amplification of specific PCR products was detected using the iQ SYBR Green Supermix (Bio-Rad, CA, USA). The RQ-PCR was performed in triplicate in a total reaction volume of 25 μl containing 1X Sybr green RQ-PCR Mix, 150 nM forward and reverse primers, and 200 ng cDNA as a template. Samples were heated for 10 min at 95 °C and were subjected to 40 cycles of PCR amplification, each cycle consisting of 15 s at 95 °C and 60 s at 60 °C. For the detection of the HTLV-1 Tax/Rex region expression, RPXPR1 and RPX4 primers were used (Kinoshita and others 1989). Within each experiment, no-template control was used to verify any contamination, and the glucuronidase beta (GUSB) housekeeping gene (NM_000181; forward primer, 5’-CAGTTCCCTCCAGCTTCAATG-3’, and reverse primer, 5’-ACCCAGCCGACAAAATGC-3’) used as a reference gene, was run in parallel. Each run was completed with a melting curve analysis to confirm the specificity of amplification and lack of unspecific products and primer dimers. Quantification was performed using the Ct comparative method. Relative gene mRNA levels were calculated as follows: 2 - [ΔCt (sample) - ΔCt (calibrator)] = 2 ^- ΔΔCt^, where ΔCt (sample) = [Ct(tax/gag gene) − Ct (GUSB)] represents the difference, in threshold cycle number, between genes and GUSB. RQ-PCR experiments were run on RT CFX96 Real-Time PCR Detection System (Bio-Rad). All primers were purchased from Primm (Milano Italy).

### NF-kB binding assay

NF-kB activation was detected by non-radioactive, Electro Mobility Shift Assay (EMSA), as previously described (Matteucci and others 2010). Briefly, aliquots of 10 μg of nuclear extracts from 5x10^6^ MT-2 cells, exposed or not to different concentrations of RES and prepared as previously described (Matteucci and others 2010), were incubated with 6 pmoles of biotin-labelled oligonucleotide probe containing the specific recognition sequence for NF-kB (AGTTGAGGGGACTTTCCCAGGC) and poly-dI-dC, to prevent unspecific reaction, in binding buffer. Binding reactions were incubated for 20 min at room temperature. The protein-DNA complexes were resolved on a 5 % native polyacrylamide gel in TBE buffer and then transferred to “Zeta-probe GT Genomic” blotting membranes (Bio-Rad). Signal from biotin-labelled probe was detected using reagents provided in the “LightShift chemiluminescent EMSA module” (Pierce, Rockford, IL) followed by exposure to autoradiography film. The NIH ImageJ software (version 1.46r, Bethesda, MD, USA) was used to evaluate densitometry of scanned films from EMSA. The integrated density of all the pixels in the area of each signal was quantified and adjusted by a background subtraction of density in an adjacent blank area of the same size. This value represents the optical density of each shift in the films. Relative density was calculated as the ratio between RES-treated and vehicle-treated samples.

### Statistics

All tests were run in triplicate or quadruplicate for each experimental condition and each experiment were repeated at least 3 times. Data were analyzed by a 1-way ANOVA with a post hoc Tukey’s test.

## Results

### Monitoring of cytokines production in MT-2 and HTLV-1 infected CD4 + lymphocytes

In order to evaluate the production of cytokines induced by an HTLV-1 infection we analyzed the kinetics of IL-17, IL-1α, IL-6 and TNF-α production in the cell culture supernatants of MT-2 cells and of peripheral T CD4+ cells infected in vitro with HTLV-1. These determinations were performed by ELISA at different time points: after 30 min, 1 h, 3 h, 6 h, 12 h, 24 h, 48 h and 72 h of culture. Each time point was tested on quadruplicate samples and expressed as pg/ml of culture supernatant.

The concentrations of IL-1α, IL-6,TNF-α and IL-17, detected in the supernatants of MT-2 cells and HTLV-1 infected CD4 + cells are described in Table 1. Data showed that all the cytokines reached higher levels in HTLV-1 infected CD4+ cells than in MT-2 cells. Although IL-17 was found in both cell cultures, with a peak around 24–48 h, it was detectable earlier in MT-2 than in HTLV-1 infected CD4 + lymphocytes. Differently from what observed for IL-17 release, IL-1α, IL-6 and TNF-α level were detectable earlier in lymphocytes than in MT-2 cell supernatants. In particular, initial amounts of both TNF-α and IL-1α in MT-2 cell were very low and reached levels comparable to those detected in HTLV-1 infected CD4+ cells supernatants after 24–48 h of culture.

**Table 1:** Proinflammatory cytokines production by MT-2 (A) and HTLV-1 infected CD4+ cells (B)

A				
Times	IL-1 α (pg/ml)^a^	IL- 6 (pg/ml)^a^	TNF-α (pg/ml)^a^	IL-17 (pg/ml)^a^
30 m	2.7 ± 0,35	29 ± 2.97	0	27.1 ± 1.8
3 h	4.1 ± 0,85	83.5 ± 4.03	1.1 ± 0.21	21.6 ± 1.75
6 h	5.1 ± 0.78	110.6 ± 14.64	2.2 ± 0.57	22.7 ± 0.8
24 h	9.3 ± 0.92	103.8 ± 12.09	7.2 ± 1.56	69.8 ± 4.2
48 h	19.9 ± 2.12	101.9 ± 13.79	20.1 ± 2.05	62.9 ± 3.6
72 h	14.4 ± 0.14	105.1 ± 15.20	18.1 ± 0.64	57.7 ± 8.2
B				
Times	IL-1 α (pg/ml)^a^	IL- 6 (pg/ml)^a^	TNF-α (pg/ml)^a^	IL-17 (pg/ml)^a^
30 m	26.5 ± 1.13	126.1 ± 4.17	28.0 ± 2.55	0
1 h	27.5 ± 0.71	118.9 ± 2.26	24.4 ± 0.64	0
6 h	25.7 ± 0.49	112.6 ± 5.23	29.3 ± 1.48	0
24 h	58.3 ± 5.37	111.4 ± 2.76	37.8 ± 0.78	26.8 ± 1.35
48 h	23.1 ± 1.98	113.7 ± 2.90	21.0 ± 0.99	49.8 ± 2.8
72 h	23.5 ± 1.63	114.1 ± 2.97	18.5 ± 1.41	45.7 ± 3.1

^a^median value ± standard deviation

In the case of HTLV-1 infected CD4 cell line, the expression of the virus was relatively stable during the ongoing of the culture and, consequently, could not affect by itself the cytokine production.

### Effect of RES on proinflammatory cytokines production

**Fig. 1:**
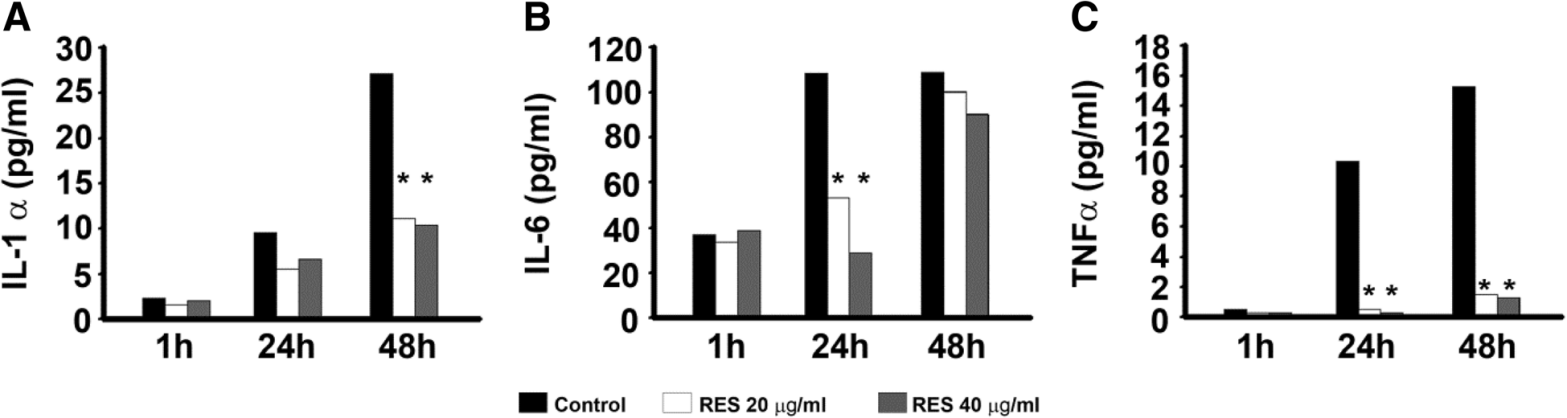
Inhibitory effect of RES on proinflammatory cytokine production by MT-2 cells. MT-2 cells were treated with 20 μg/ml and 40 μg/ml of RES for 1 h, 24 h and 48 h and cytokine production was assessed by ELISA. **a** Effects on IL-1α production. **b** Effects on IL-6 production. **c** Effects on TNF-α production. CTR = cells treated with DMSO alone. Each experiment was performed in quadruplicate. The results are expressed as mean pg/ml. Error bars are omitted (the standard error SEM were less than 10 %). * *P* < 0.01

The inhibitory effects of RES on IL-1-α, IL-6 and TNF-α release in MT-2 cells, after treatment with 20 μg/ml and 40 μg/ml of RES for 1 h, 24 h and 48 h in comparison with untreated cells, are shown in Fig. 1. According to the length of treatment: a) no inhibition after 1 h; b) significant, dose dependent reduction of IL-6 and TNF-α (100 %) after 24 h, at both concentrations (*P* < 0.01); c) significant reduction of IL-1α and TNF-α production, at both concentrations (*P* < 0.01), and recovery of IL-6 production, after 48 h. The inhibitory effect on TNF-α was visible also at the lowest concentrations of RES (from 0.612 to 2.5 μg/ml) and lasted until 48 h (data not shown).

**Fig. 2:**
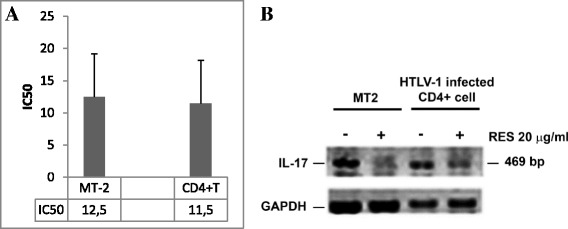
Inhibitory effect of RES on IL-17 production. PANEL **a** show the **i**nhibitory effect of different concentrations of RES on IL-17 production. MT-2 cells and HTLV-1 infected CD4+ T cell cultures were treated with RES at 0.612,1.25,2.5,5,10,20 and 40 μg/ml for 48 h of culture. IL-17 production was assayed by ELISA and the results are expressed as IC50, i.e., the concentration of RES where the IL-17 production is reduced by 50 %. The bars represent the confidence limits. PANEL **b** show the Effect of RES on IL-17 transcription in MT-2 cells and HTLV-1 infected CD4+ cells. Cells were treated with RES at 20 μg/ml for 48 h and IL-17 transcription was assessed by RT-PCR. The image refers to visualization of IL-17 mRNA expression in DMSO treated MT-2 cells, RES-treated MT-2 cells, DMSO treated HTLV-1 infected CD4+ cells and RES-treated HTLV-1 infected CD4+ cells

Levels of IL-17 were evaluated in culture supernatants from MT-2 cells and HTLV-1 infected CD4+ cells after 48 h of addition of RES at different concentrations (0.612, 1.25, 2.5, 5, 10, 20 and 40 μg/ml), cultures with DMSO alone were prepared as negative controls. Results, expressed in terms of IC50 inhibitory dose, i.e. the concentration of RES reducing 50 % of IL-17 production, are illustrated in Fig. 2 Panel **a**. RES induced a dose-dependent inhibition on IL-17 production, reaching 100 % of inhibition at 20 μg/ml in HTLV-1 infected CD4 + cells, and at 40 μg/ml in MT-2 cells (data not shown). In any case the IC50 value is comparable both in MT-2 cells and HTLV-1 infected CD4 + .

**Fig. 3:**
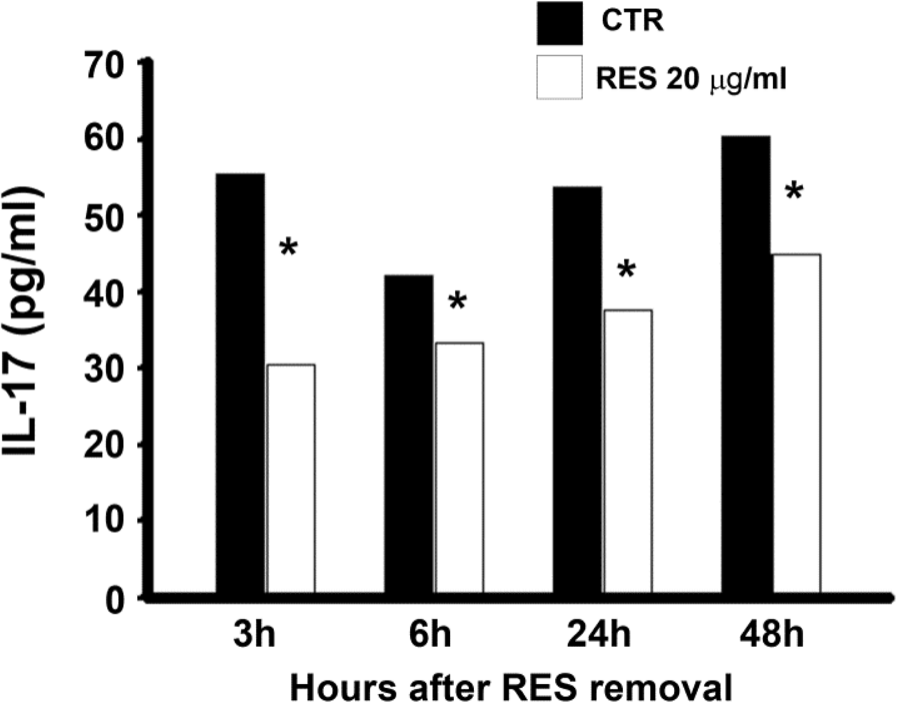
Reversibility of the inhibitory effect of RES on IL-17 production by MT-2 cells. MT-2 cells were treated with 20 μg/ml of RES for 48 h. IL-17 production has been assessed by ELISA in the supernatants at 3 h, 6 h, 24 h and 48 h of culture after RES removal. CTR = cells treated with DMSO alone. Each experiment was performed in quadruplicate. The results are expressed as mean pg/ml. Error bars are omitted (the standard error SEM were less than 10 %). * *P* < 0.01

The recovering from the inhibitory effect of RES (20 μg/ml) on IL-17 production was evaluated in supernatants of MT-2 cells at 3 h, 6 h, 24 h and 48 h of culture, after RES removal. The IL-17 production was still significantly lower in treated cell cultures than in controls (*P* < 0.01) and this inhibitory effect lasted at least 48 h (Fig. 3).

### Effect of RES on the expression of IL-17 mRNA

The effect of RES was evaluated on IL-17 mRNA using RT-PCR analysis. Results from a representative experiment are shown in Fig. 2 Panel **b**. RES at concentration of 20 μg/ml was clearly capable to down-regulate in 48 h the IL-17 mRNA in MT-2 cells and in HTLV-1 infected CD4+ lymphocytes. Accordingly with data showing the inhibition of IL-17 cytokine production (Fig. 2 Panel **a**), this result suggested that RES was most likely able to interfere with IL-17 release also at transcriptional level.

### Effects of RES on NF-kB activity in MT-2 cells

**Fig. 4:**
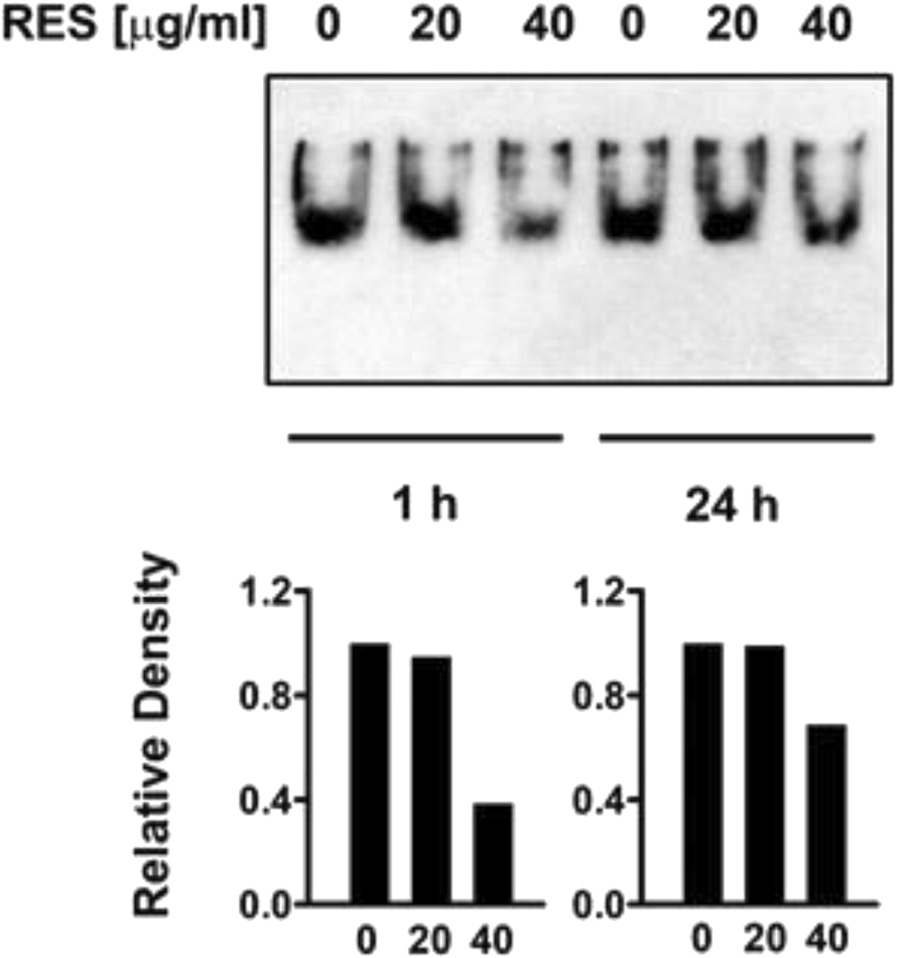
Suppressive effect of RES on NF-kB activation in MT-2 cells. MT-2 cells were either treated with vehicle or treated with 20 μg/ml or 40 μg/ml RES and after 1 h and 24 h, NF-kB complex activation was assayed by non-radioactive EMSA. The upper panel refers to the autoradiography film while the lower panels represent the relative density of the bands with respect to vehicle treated samples, from one representative experiments of the two performed with similar results

**Fig. 5:**
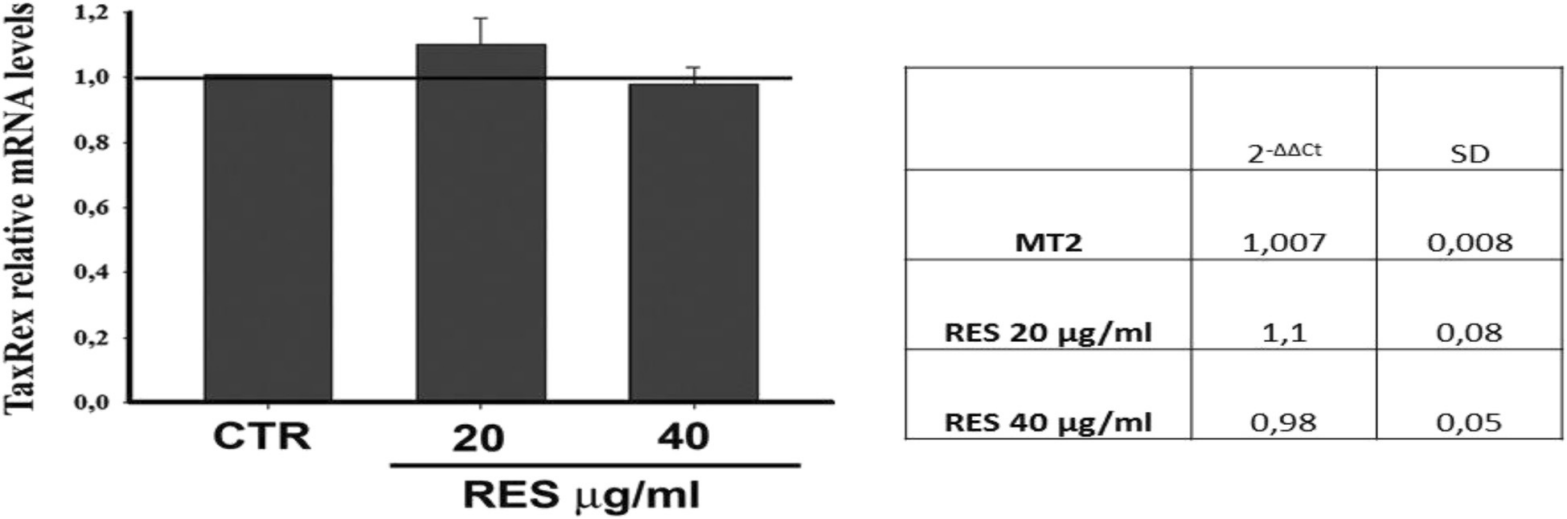
Effect of RES on HTLV-1 Tax/Rex expression. MT-2 cells were either treated with vehicle (CTR) or with 20 μg/ml or 40 μg/ml RES and after 24 h Tax/Rex expression was evaluated through real-time PCR (RQ.PCR). The values were expressed as TaxRex relative mRNA levels in treated cells with respect to vehicle treated samples

With the aim of better understanding the mechanism responsible for the inhibitory effect of RES on IL-17 as well as its effect on the other cytokines assessed, we verified whether RES might directly affect the activation of the NF-kB complex. To this end, HTLV-1 chronically infected MT-2 cells were either treated with 20 μg/ml or 40 μg/ml of RES or exposed to vehicle (as negative control) and NF-kB activation was assessed as DNA-binding activity in nuclear lysates, harvested after 1 and 24 h from RES treatment, respectively, using a non-radioactive EMSA (Fig. 4). Equal amounts of nuclear extracts derived from equal amounts of viable cells from control or RES treated samples, were utilized, in order to avoid any bias related to differences in total cell number. A clear dose-dependent inhibition of NF-kB binding activity was found after RES treatment. The suppressive effect was particularly evident at 40 μg/ml and early after exposure to the compound (at 1 h after treatment), as shown by the quantitative determination of the density of the bands (Fig. 4). Conversely RES at the concentration of 20 and 40 μg/ml, did not induce downregulation of HTLV-1 Tax/Rex expression (see Fig. 5).

## Discussion

In the present study we investigated the effects of RES on proinflammatory cytokines production from MT-2 cell line and from peripheral healthy donors CD4+ lymphocytes infected *in vitro* by HTLV-1. HTLV-1 infection is associated with systemic immune–mediated inflammatory diseases whose hallmarks include increased levels of IFN-γ, TNF-α, and IL-6 [2, 11, 25] and remarkable tissue damage [1]. The increased production of proinflammatory cytokines and the expansion of autoreactive T cells observed in HTLV-1–infected patients appear, at least in part, due to the lack of regulatory T cell function and a decreased ability of IL-10 and TGFβ to regulate the immune response [26]. In fact, IL-17 mRNA is highly expressed in HTLV-1-infected cells [13]. In infected CD4^+^ T cells, the Tax viral protein is capable of up-regulating the expression of IL-17, which, in turn, has the ability to stimulate the production of other inflammatory cytokines and chemokines, including IL-6, IL-8, GM-CSF and MCP-1 [26]. In previous studies we found that RES is able to counteract the production of IL-17 and Th1/Th2 cytokines in an inflammatory cell model in vitro [17, 18]. Due to these anti-inflammatory and immunomodulating properties [27]. RES might represent a good candidate for chemoprevention strategies or for use in combination therapy in HTLV-1 related diseases.

In the present study we firstly analyzed the profiles of inflammatory cytokine production *in vitro* in two different cell systems, including the HTLV-1 transformed continuous MT-2 cell line and short term culture of CD4+ lymphocytes from healthy donors immortalized in our laboratory by HTLV-1.

We showed that HTLV-1 infected cells produce high amounts of inflammatory cytokines (Table 1), such as IL-1α, IL-6 and TNF-α, and that RES exerts an inhibitory effects on the HTLV-1 induced production of these cytokines (Fig. 4). In particular, TNF-α was strongly inhibited. Further experiments confirmed that RES can inhibit TNF-α and IFN-γ in vitro even at very low concentrations (about 1 μg RES/ml and 5 μg/ml RES respectively, data not shown). The exact role of an individual cytokine in the complex inflammatory milieu induced by HTLV-1 infection cannot be easily defined. It is well known that both TNF − α and IFN-γ exert a synergistic effect on IL-17-induced production of IL-6 [28]. IL-17 appears to play a key role in the cytokine circuitry induced by HTLV-1 infection, as shown in HAM/TSP patients [29]. However, conflicting results have been recently shown on the possible role of Th17 T cells and IL-17 production in HTLV-1 infected patients [30].

Our results showed that both HTLV-1 infected cell models spontaneously produce IL-17 in vitro although with different kinetics. In the MT-2 cell line the release of IL-17 was detected as early as 30 min, whereas in HTLV-1 infected CD4+ T cells it was found after 12 h of culture (Fig. 1). Both cell types reached peak levels of IL-17 production after 24 or 48 h in culture, respectively. These data are consistent with previous observations in HTLV-1-infected T cells showing that IL-17 mRNA is induced in association with Tax expression [13].

With the aim of investigating the effect of RES, IL-17 production was assessed in MT-2 and in HTLV-1 infected CD4+ T cells in the presence or in the absence of RES and found that in the presence of RES IL-17 is strongly reduced reaching a 100 % inhibition both in MT-2 cells treated with RES 40 μg/ml (Fig. 2 Panel a) and in CD4+ infected T cells, treated with 20 and 40 μg/ml of RES (Fig. 2 Panel b), respectively. In order to exclude that the low levels of cytokines might depend on cytotoxic effects of RES, although in our experimental conditions the concentrations of RES were well below the toxic concentrations cell viability was assessed after RES incubation by Trypan blue exclusion test (data not shown).

Figure 3 shows that the inhibitory effect of RES on IL-17 production in MT-2 cells was still present up to 48 h after its removal at both protein and RNA levels (Fig. 5) in both cell models. Thus, these data indicate that the inhibitory effect of RES on IL-17 involve, at least in part, biochemical pathways independent from those governing the cell viability.

Molecular pathways presumably involved in the transcriptional activation of the IL-17 gene in HTLV-1-infected cells include the Tax protein, which in turn depend on the CREB/ATF pathway and the interferon regulatory factor 4 (IRF4), a transcription factor that regulates IL-17 and IL-9, which is over-expressed in HTLV-1-infected cells with a Th17 phenotype [13]. Moreover, a key role for the retinoic orphan receptor RORC, a Th17 transcription factor, has been also suggested in the regulation of IL-17 expression [13, 30]. The effect of RES on the above described molecular pathways is currently unknown and further studies are needed to understand this aspect. On the other hand, IL-17 receptor signaling induces the activation of NF-kB, which, in turn, results in elevated levels of inflammatory cytokines [31].

Recent studies suggest that NF-kB may play a key role in mediating RES effects since RES-induced inhibition of inflammatory cytokine production is achieved by suppressing NF-kB activation [32], although it has been argued that RES might hit multiple targets [33].

In the present study, we found that RES down-regulate IL-17 in MT-2 cells through an inhibition of NF-kB activation (Fig. 4). Although it is not possible, at the moment, to trace the cascade of events triggered by RES and leading to the down-regulation of IL-17 and other inflammatory cytokines in HTLV-1 infected cells, nevertheless, based on our results and on existing literature [34-36], it appears conceivable that a strict connection exists between RES-induced inhibition of the inflammatory cytokine circuitry and the down regulation of NF-kB.

In addition, it is well recognized that the HTLV-1 Tax protein primarily targets the IkB kinase complex, resulting in persistent activation of NF-kB and upregulation of its responsive genes that, in turn, are crucial for T cell survival and cell cycle progression [34]. It has been shown that RES inhibits NF-kB and modulates IkB kinase and this may contribute to the inhibition of inflammatory cytokines [35, 36]. Thus, compelling evidence suggest that the inhibition of NF-kB by RES represents a key step for the downregulation of inflammatory cytokines. However, whether this depend or not on IL-17, is still an open question.

On the other hand, the failure of RES in suppressing IL-6 production could be due to the fact that in addition to NF-kB mediated transcription, the IL-6 gene can be activated by alternative mechanisms [37].

RES has been shown to inhibit the replication of herpes viruses as well as the growth of EBV-immortalized cell lines and polyomavirus [38, 39]. In HTLV-1 infection, RES has been shown to inhibit the growth of virus-infected cell lines by down-regulating survivin expression [40]. In our study the treatment of MT-2 cells with RES was unable to affect HTLV-1 expression. Similarly Tang et al. [41] have shown that low Res concentrations did not significantly inhibited virus expression in MT-2 cells in vitro. Conversely they reported that increasing amount of Res inhibited Tax-mediated LTR transactivation in SIRT-1 overexpressing HEK293 cells (Tang et al.). These studies suggest that Res is able to abrogate Tax transactivation only under condition of LTR overexpression, in presence of SIRT-1 deacetylase, while in physiological conditions the inhibitory effect of Res on HTLV-1 expression is rather cell type dependent [41].

More recently, it has been shown that RES at high concentration (50–100 μM) had potent antiproliferative effect on ATL cells [42].

In our study we found that that although RES did not inhibit HTLV-1 expression (data not shown), and beside an anti-proliferative effect toward HTLV-I infected cells exerted at high concentrations, at lower concentrations RES can exert an evident, dose-dependent anti-inflammatory activity. These results strongly suggest RES can either act on cell growth and the inflammatory circuitries through distinct mechanisms.

## Conclusions

Anti-retroviral agents used for HIV have partially improved the prognosis of ATL, but little effect has been shown on HAM/TSP [43]. Moreover, an effective therapy for HTLV-1 associated diseases still represent an unmet need. Results of the present study suggest that RES can exert an anti-inflammatory activity on HTLV-1 infected cells at low doses, in association with a suppressive effect on NF-kB activation. Taken together, these results suggest that RES, or its derivative, given also its safety profile in clinical trials [44, 45], might represent a promising therapeutic agent, in combination with both anti-inflammatory and/or antiretroviral drugs, for the treatment of HTLV-1-related diseases, including HAM/TSP.
